# Histologic Correlation With Magnetic Resonance Imaging for Benign and Malignant Lipomatous Masses

**DOI:** 10.1080/13577149778272

**Published:** 1997-12

**Authors:** Bruce T. Rougraff, Mark Durbin, Jackie Lawerence, Kenneth Buckwalter

**Affiliations:** 1 Indiana University School of Medicine Indianapolis IN USA; 2 702 Barnhill Drive Room 1134 Indianapolis IN 46202-5111 USA

## Abstract

*Purpose/results*. We evaluated the diagnostic accuracy of magnetic resonance imaging (MRI) for 46 consecutive patients with lipomatous soft tissue tumors prior to biopsy and resection. Twenty-eight patients had benign lipomas and 18 had liposarcomas. Clinical differences between thdse patients with benign disease and those with malignant lesions were average age at the time of presentation (49 years for benign vs 62 years for malignant, *p* < 0.001) and average length of symptoms prior to resection (64 months for benign versus 38 months for malignant, *p* = 0.01). MRI characteristics associated with benign disease included: smaller tumor size (9.4 cm average greatest dimension for benign lesions vs 13.4 cm for malignant masses, *p* = 0.022); a mass with a uniformly homogeneous signal (*p* = 0.0003); a mass with homogeneous high T1 and T2 signals and a low short-time-inversion-recovery (STIR) signal comparable to normal fat (*p* < 0.0001). This last signal pattern was not seen in malignant lesions (0/18) and was present in almost all benign lipomas (25/28). The usual MRI descriptions of soft tissue masses such as infiltrating vs encapsulating, deep vs subcutaneous and septated vs non-septated were not helpful predictors of malignancy in this series. Needle biopsies of lipomatous masses with heterogeneous signals on MRI resulted in inaccurate diagnoses due to sampling error in 5/9 patients.

*Discussion*. A carefully planned and performed MRI study of lipomatous masses can accurately predict a benign lipoma whenever a homogeneous high T1 and T2, as well as a low STIR, signal is present. However, a mass with any other signal characteristics must be biopsied carefully in order to make an accurate diagnosis.


*Sarcoma* (1997) **1**, 175–179

CARFAX

**ORIGINAL ARTICLE**

### Histologic correlation with magnetic resonance imaging for benign and malignant lipomatous masses

BRUCE T. ROUGRAFF, MARK DURBIN, JACKIE LAWERENCE & KENNETH BUCKWALTER

*Indiana University School of Medicine, Indianapolis, IN, USA*

**Abstract**

*Purpose/results.* We evaluated the diagnostic accuracy of magnetic resonance imaging (MRI) for 46 consecutive patients with lipomatous soft tissue tumors prior to biopsy and resection. Twenty-eight patients had benign lipomas and 18 had liposarcomas. Clinical differences between those patients with benign disease and those with malignant lesions were average age at the time of presentation (49 years for benign vs 62 years for malignant, $p < 0.001$) and average length of symptoms prior to resection (64 months for benign versus 38 months for malignant, $p = 0.01$). MRI characteristics associated with benign disease included: smaller tumor size (9.4 cm average greatest dimension for benign lesions vs 13.4 cm for malignant masses, $p = 0.022$); a mass with a uniformly homogeneous signal ($p = 0.0003$); a mass with homogeneous high T1 and T2 signals and a low short-time-inversion-recovery (STIR) signal comparable to normal fat ($p < 0.0001$). This last signal pattern was not seen in malignant lesions (0/18) and was present in almost all benign lipomas (25/28). The usual MRI descriptions of soft tissue masses such as infiltrating vs encapsulating, deep vs subcutaneous and septated vs non-septated were not helpful predictors of malignancy in this series. Needle biopsies of lipomatous masses with heterogeneous signals on MRI resulted in inaccurate diagnoses due to sampling error in 5/9 patients.

*Discussion.* A carefully planned and performed MRI study of lipomatous masses can accurately predict a benign lipoma whenever a homogeneous high T1 and T2, as well as a low STIR, signal is present. However, a mass with any other signal characteristics must be biopsied carefully in order to make an accurate diagnosis.

**Key words:** *magnetic resonance imaging, lipoma, liposarcoma, benign, malignant.*

### Introduction

Magnetic resonance imaging (MRI) has greatly enhanced our ability to analyze the anatomical relationships and some internal characteristics of soft tissue masses. MRI of extremity soft tissue masses can show tissue contrast and tumor margins that are superior to images obtained with other modalities (i.e. computed tomography, ultrasound or angiography).[1–4] Therefore, MRI is frequently the imaging procedure of choice for the evaluation of soft tissue neoplasms. Unfortunately, many large soft tissue tumors have indistinct (MRI) characteristics and the MRI cannot determine whether the tumour is benign or malignant.[5,6].

Normal fat tissue has a characteristic MRI appearance. Fat has a high signal on T1-weighted images, a high signal on T2-weighted images and a low signal on short-time-inversion-recovery (STIR) images.[2,7] Furthermore, the appearance of the subjacent subcutaneous fat acts as a control for the presence or absence of normal fat within a lipomatous tumor. It is a deviation in appearance from normal fat that raises a concern about the benign nature of a lipomatous tumor. This relationship remains constant regardless of pulse sequence or imaging parameters. Because every patient has normal subcutaneous fat tissue, each MRI study has an 'internal control' for comparison with a suspected soft tissue mass. Sarcomatous tissue usually has non-fatty elements which are represented by a lower T1, higher T2 and a higher STIR signal than the surrounding normal subcutaneous fat. Most other non-fatty soft tissue neoplasms (benign and malignant) have these same MRI characteristics making this a very non-specific pattern.

Benign lipomas represent the most common soft tissue mass[8] and liposarcomas are the second most common soft tissue malignancy in adults.[9] Because the surgical management of these lesions varies greatly depending on the histological diagnosis, it is helpful to define the MRI characteristics of benign and malignant lipomatous masses. We chose to evaluate the clinical features at the time of referral, the predictive value of the MRI signal characteristics, and the biopsy results in patients with benign benign and malignant lipomatous masses treated at our institution.

Correspondence to: B. T. Rougraff, 702 Barnhill Drive, Room 1134, Indianapolis, IN 46202-5111, USA. Fax: +1 317 274 3702.

1357-714X/97/030175-05 $9.00 © 1997 Carfax Publishing Ltd

### Patients and methods

Forty-six consecutive patients with lipomatous soft tissue masses were evaluated in a blind fashion over a 3-year period using MRI prior to biopsy and excision. These MRI studies were examined by one co-author (MD) who was blind to the diagnosis and was not involved in the care of the patents. Data were tabulated for the anatomic location, size and depth of the mass. The size of the mass was recorded in centimeters to describe length, width and height. The overall largest dimension for the mass was used for size analysis. Depth of the mass was scored as subcutaneous, intermuscular or intramuscular. Each lesion was also characterized as either encapsulated or infiltrating, and it was noted whether septations were present within the lesion. Every patient underwent a complete study of their lesion that involved multiple plane analysis with T1-weighted and T2-weighted sequences. Twenty-seven of the 46 patients also underwent a STIR sequence. For clinical imaging of soft tissue masses, STIR sequences are selected resulting in nulling of the signal from fat within the image. These STIR images, which are closely related to the conventional spin echo sequences, are widely used in musculoskeletal applications.[7]

All MRI studies were assessed for the signal intensity of the mass based on T1-weighted, T2-weighted and STIR images, along with the signal intensity of the mass compared to uninvolved subcutaneous fat on T1-weighted, T2-weighted and STIR images. Each category was recorded as high, intermediate or low signal. When compared to subcutaneous fat, masses were scored as greater than, less than or equal to the signal intensity of the subcutaneous fat surrounding the mass. Each mass was also described as having a homogeneous or heterogeneous signal. Following initial clinical evaluations and radiographic analysis, a needle, incisional or excisional biopsy was performed at the discretion of BTR.

All biopsy material and resected specimens were evaluated by the senior author and a faculty member of the pathology department. The final diagnosis was based on a thorough pathologic study of the entire specimen.

### Results

### *Clinical findings*

Of the 46 patients with lipomatous masses (Table 1), there were 24 males and 22 females with an average age of 54 years (range 29–87 years). There was a total of 28 benign and 18 malignant lipomatous masses. The duration of symptoms prior to referral averaged 54 months (range 1–240 months). The masses were located in the upper extremity in 13 patients and in the lower extremity in 27 patients. Three masses were paraspinous and three others were flank masses. The average age of patients with a benign mass at the time of treatment was lower than that of patients with liposarcomas (49 years, range 29–75 years, as compared to 62 years, range 48–87 years). This represented a statistical difference ($p < 0.001$). The average duration of symptoms prior to referral for patients with benign disease was 64.1 months which was longer than for the malignant group (38.0 months, $p = 0.010$).

### *MRI findings*

Several standard MRI descriptive terms were assessed to test whether they were predictive of malignant vs benign lipomatous masses. Of the 46 masses (Table 2), 30 were described as encapsulated and 16 infiltrating. Twenty-one lipomas were described as encapsulated and seven were infil-

**Table 1.** *Clinical differences of 46 patients with benign and malignant lipomatous masses*

| Parameter | Benign | Malignant |
|---|---|---|
| Number of masses | 28 | 18 |
| Male | 14 | 10 |
| Female | 14 | 8 |
| Average age (years) | 49 | 62 |
| Length of symptoms (months) | 64 | 38 |

The clinical information of 46 patients who were studied using MRI followed by biopsy and complete excision is depicted in table format comparing data of benign vs malignant lesions. The average age was the age at the time of treatment. The length of symptoms was the patient's report of how long they were aware of the mass prior to treatment.

**Table 2.** *MRI anatomic characteristics of 46 lipomatous masses*

| Parameter | Benign | Malignant | Significance |
|---|---|---|---|
| Number of patients | 28 | 18 | |
| Depth | | | $p = 0.064$ |
| Subcutaneous | 10 | 2 | |
| Intramuscular | 13 | 11 | |
| Intermuscular | 5 | 5 | |
| Size (cm) | 9.4 | 13.4 | $p = 0.002$ |
| Encapsulated | 21 | 9 | $p = 0.082$ |
| Infiltrating | 7 | 9 | |
| Homogeneous | 25 | 7 | $p = 0.0003$ |
| Heterogeneous | 3 | 11 | |
| Septated | 13 | 8 | $p = 0.895$ |

The traditional MRI anatomic characteristics for 46 patients with lipomatous masses are depicted in table format and compared as benign vs malignant lesions. The size data were recorded from the MRI in three dimensions but only the average of the largest dimension (in cm) is reported here.

**Table 3.** *MRI signal characteristics comparing benign vs malignant lesions*

| Parameter | Benign | Malignant | Statistics |
|---|---|---|---|
| Homogenous: high T1, high T2, low STIR | 25/28 | 0/18 | $p < 0.001$ |
| Heterogenous: T1, T2, STIR | 3/28 | 11/18 | $p < 0.001$ |
| Homogenous: low T1, high T2, high STIR | 0/28 | 7/18 | $p < 0.001$ |

Three patterns of MRI signal characteristic are reported in table format to show the differences between benign and malignant lesions.

trating as compared to nine encapsulated liposarcomas and nine infiltrating. Evidence of encapsulation vs infiltration on the MRI was not predictive of tumor grade ($p = 0.082$). Twenty-four of the masses were described as intramuscular, ten intermuscular and twelve subcutaneous. MRI characteristics of compartmentalization of the mass did not help to distinguish between benign and malignant lesions ($p = 0.130$). Masses that were deep to the muscle fascia were malignant in 16/34 cases and subcutaneous lesions were malignant in 2/12 cases. Depth of the lesion was also not strongly predictive of malignancy ($p = 0.064$). Twenty-one of the 46 masses had septations. Thirteen of the 28 benign masses were found to have septations vs 8/18 liposarcomas; this offered no clinical help ($p = 0.895$).

Liposarcomas tended to be larger than benign lipomas on MRI imaging. The average measurement of the largest dimension of the benign mass was 9.4 cm (range 4–21 cm) and 13.4 cm (range 3–24 cm) for patients with liposarcomas ($p = 0.022$).

Certain MRI signal characteristics were studied for their predictive potential for liposarcomas vs lipomas (see Figs 1–3). Homogeneous high T1 and T2 and low STIR MRI signal characteristics were present in all 25 benign lipomas. Of the remaining 21 masses that did not have these characteristics, 18 were malignant (Table 3). This was highly statistically significant ($p < 0.001$). The three patients who had benign masses, yet had a heterogeneous signal simulating a malignancy, were spindle cell variant of a benign lipoma, a fibrolipoma and a recurrent atypical lipoma.

### *Pathologic findings*

The final pathologic diagnosis was benign lipoma in 28 patients which consisted of 15 intramuscular lipomas, 10 subcutaneous lipomas, one deep fibrolipoma, one spindle cell lipoma and one benign, deep atypical lipoma. Of the liposarcomas (18 masses), there were four myxoid, four dedifferentiated, two pleomorphic, four well differentiated, one round cell and three low-grade liposarcomas.

Careful study of the benign lipomas revealed no evidence of primative lipoblasts or frank malignant features. However, the linear strands that were seen on MRI were also identified on histologic evaluation of the benign lipomas. These linear strands appeared to be normal vessels and fibrous bands. It was difficult to tell whether the fibrous bands were produced by the tumors or were portions of normal fascia that had been incorporated into the mass as it grew, displacing the surrounding muscle and neurovascular structures. Soft tissue masses are more difficult than bone lesions to carry out exact MRI to pathologic mapping, since soft tissue lesions change shape with manipulation. However, areas of dedifferentiation and other grossly observable changes within the masses could be correlated with the MRI studies.

Although not a primary focus of this research, we evaluated the accuracy of limited biopsy samples compared to the final diagnosis based on the entire specimen. There was a total of nine needle biopsies of which five were incorrectly diagnosed histologically. Of these five, one benign lesion was diagnosed as an atypical lipoma from needle biopsy with a final pathology diagnosis of spindle cell lipoma. Four malignant lesions were misdiagnosed by needle biopsy. Three of these had erroneous benign diagnoses that were corrected after an incisional biopsy and later resection. The fourth biopsy was erroneously interpreted as low-grade malignancy when in fact the lesion contained areas of high-grade dedifferentiation. This problem of inaccurate limited biopsies occurred only in patients with masses that had heterogeneous signals on MRI, strongly suggesting that sampling errors had occurred. Incisional biopsies were performed in seven of these patients and were correctly interpreted in all of them.

### Discussion

Certain clinical clues have traditionally been used to aid in the diagnosis between benign and malignant soft tissue masses. Malignant soft tissue masses are typically larger than 5 cm, are located deep to the muscle fascia, have a short history of rapid enlargement and have an infiltrating or poorly encapsulated appearance on radiographic images.[8] Unfortunately, none of these characteristics was universally predictive of malignancy in our series. We found that even benign lipomas can become quite large, comparable to liposarcomas, although liposarcomas do tend to

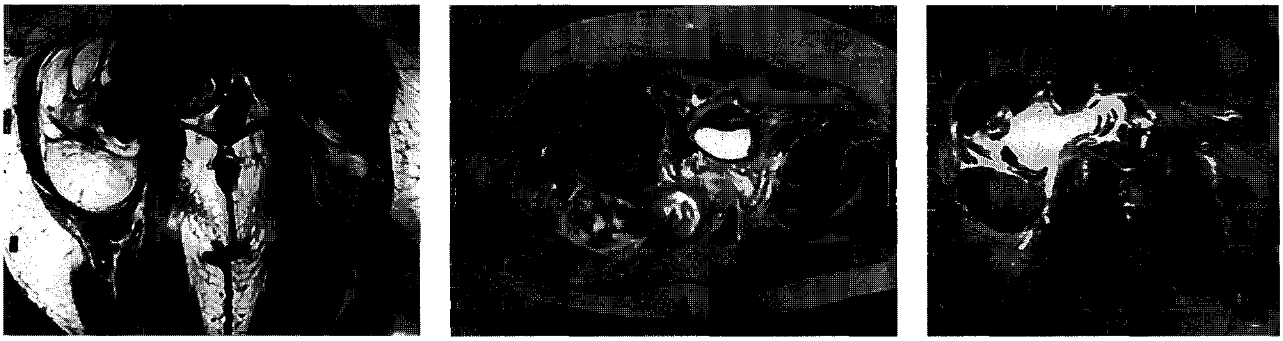

(a) (b) (c)

**Fig. 1.** *(a) A 65-year-old patient presented with a large, deep buttock mass that is seen here on a T1-weighted, coronal MRI study. There is a large mass which is deep to the gluteus maximus muscle fascia and has a tissue signal which is mostly equivalent to the normal subcutaneous fat. However, the mass extends into the greater sciatic foramen and, in this region, the mass has a dark T1 signal very different from normal fat. (b) The same patient with an axial cut T2-weighted image that shows a high signal intensity different from the rest of the lesion. (c) The same patient studied with a coronal STIR sequence in the same plane as the T1 shown in Fig. 1(a). Here the segment of the tumor within the sciatic notch is clearly of higher signal intensity than normal fat. The pathologic evaluation of this area revealed a high-grade, dedifferentiated liposarcoma.*

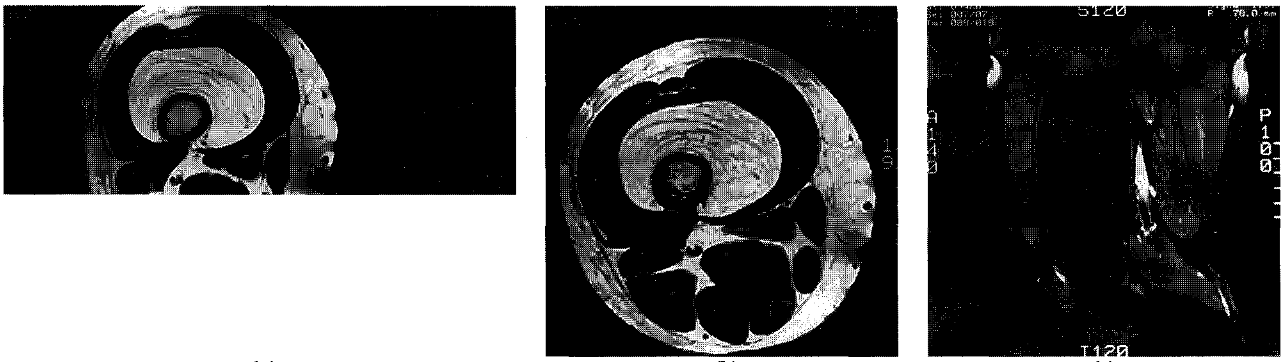

(a) (b) (c)

**Fig. 2.** *(a) A T1-weighted axial MRI study of a 42-year-old female with a slowly enlarging mass on her thigh. The T1 image shows a large, deep, high-signal mass with septations that completely encases the femur. (b) This T2-weighted image shows similar findings as the axial T1 images seen in Fig. 2(a). The mass is similar to the subcutaneous fat with the exception of the septations that are present. (c) A sagital STIR image of the same lesion. This image shows a very dark and homogeneous signal mass that is equivalent to subcutaneous fat and darker than normal muscle. Note that the septations seen on T1 are not bright on STIR. Thorough pathologic evaluation revealed a benign lipoma without evidence of atypia. She underwent a marginal excision and is disease free 2 years after excision. MRI predicts that this is a benign lipoma because of the homogeneous signal, particularly on STIR sequences.*

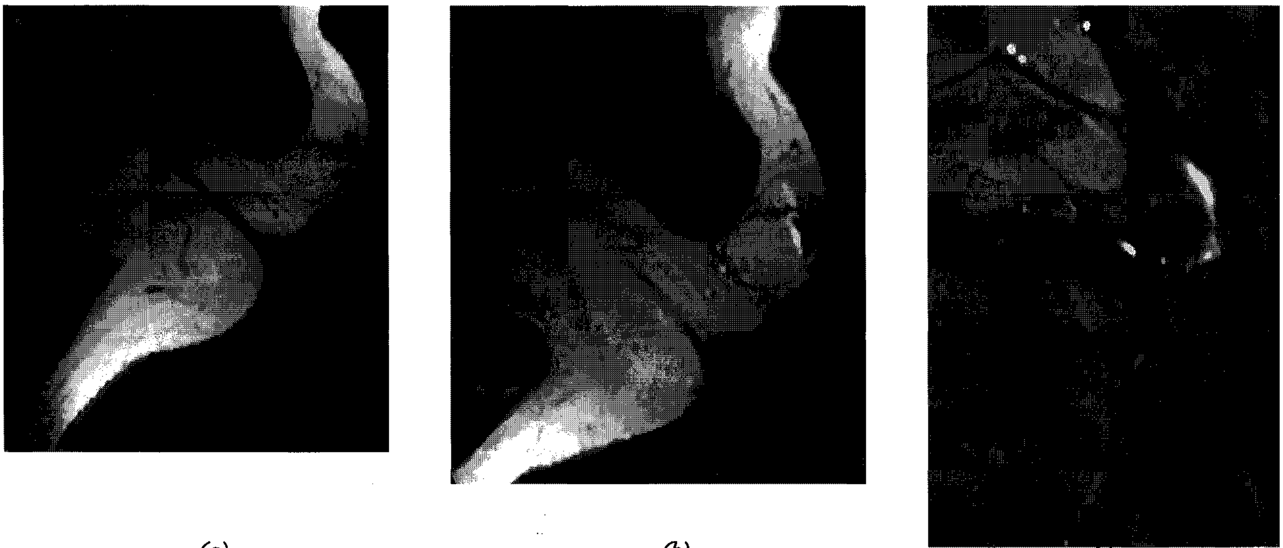

(a) (b) (c)

**Fig. 3.** *(a) A T1-weighted axial image of a 50-year-old man with a rapidly enlarging subcutaneous mass. The T1 signal is heterogeneous, but mostly equivalent in characteristics to the surrounding fat. (b) A T2-weighted image of the same lesion showing similar signal characteristics to fat, except for a small eccentric region of high signal. (c) A STIR study of the same lesion showing significant areas of high intensity that is very different from the surrounding fat. This difference is most obvious on this STIR sequence. The lesion was biopsied and excised and the pathologic diagnosis was benign, spindle cell lipoma. This heterogeneous presentation is similar to how a liposarcoma could appear. MRI cannot make this determination when there is a heterogeneous signal present.*

be larger on average. Likewise, we have shown that for lipomatous masses, depth is not always a reliable indicator of malignancy. Septations and encapsulation of lipomatous masses were also poor indicators of malignancy with 50% of the liposarcomas showing encapsulation and 46% of the benign lipomas having septations.

We have shown that certain MRI signal characteristics are very accurate at diagnosing benign lipomas. The sensitivity in predicting a benign lipoma was 89% and specificity reached 100% in this study. The positive predictive value was 100% for all 25 patients with a homogeneous high T1, high T2 and low STIR signal (comparable to normal fat). All of the homogeneous masses with low T1, high T2 and high STIR signals (contrasting with normal fat) were malignant. However, this latter MRI pattern is not diagnostic of liposarcomas since large neurilemmomas, neurofibromas, myxomas and other types of soft tissue benign and malignant neoplasms can have this MRI appearance.[9–11] A biopsy would be necessary to diagnose a mass with this MRI pattern accurately. The third MRI pattern of heterogeneous signals represents a diagnostic problem. Although most of these masses were liposarcomas (11/14 patients), needle biopsies misdiagnosed the lesions more than half of the time due to sampling errors of the heterogeneous mass. This would have resulted in undertreatment of four liposarcomas in this series of patients if incisional biopsies had not been performed prior to resection.

In conclusion, if the MRI study completely images the mass in multiple planes, using thin cuts and with T1, T2 and STIR sequences, and the mass has a homogeneous signal comparable to normal fat (high T1, high T2 and low STIR), the surgeon can assume that this is a benign lipoma. Observation of the patient without a biopsy is supported by our findings. A complete excision without contamination of the pseudo-capsule of this lesion would be indicated if the lesion is sufficiently symptomatic. However, if a patient has a mass with a heterogeneous MRI characteristic signal, the lesion is likely to represent a liposarcoma. A needle biopsy of a heterogeneous mass interpreted as benign should then undergo an incisional biopsy by a surgeon who is experienced in oncologic surgery because a sampling error may have occurred. If a biopsy is to be performed of a mass with a heterogeneous signal, a sample of an area of the mass with low T1, high T2 and high STIR should be obtained for the most accurate diagnosis. If the mass has a homogeneous signal that does not compare to fat on any sequence, MRI can only predict that it is not a typical benign lipoma. This pattern can be seen with sarcomas as well as several types of benign masses. Treatment of these lesions should be based on an accurate biopsy.

### References

1 Berquist TH, Ehman RL, King BF, *et al.* Value of MR imaging in differentiating benign from malignant soft tissue masses. *Am J Radiol* 1990; 155:1251–5.

2 Dooms GC, Hricak H, Sollitto RA, *et al.* Lipamatous tumors and tumors with fatty component: MR imaging potential and comparison of MR and CT results. *Radiology* 1985; 157:479–83.

3 Petasnick JP, Turner DA, Chaters JR, *et al.* Soft tissue masses of the locomotor system: comparison of MR imaging with CT. *Radiology* 1986: 160:125–33.

4 Roth D, Widelec J, Ramon F, *et al.* Adipose tumors of the soft tissues. *J Br Radiol* 1992; 75:321–6.

5 Crim JR, Seeger LL, Yao L, *et al.* Diagnosis of soft tissue masses with MR imaging: can benign masses be differentiated from malignant ones? *Radiology* 1992; 185:581–6.

6 Kransdorf MJ, Jelinek JS, Moser RP, *et al.* Soft-tissue masses: diagnosis using MR imaging. *Am J Radiol* 1989; 153:541–47.

7 Murphy WD, Hurst GC, Duerk JL, *et al.* Atypical appearance of lipomatous tumors on MR images: high signal intensity with fat-suppression STIR sequences. *J Magnetic Res Imaging* 1991; 1:477–80.

8 Rydholm A, Berg NO. Size, site and clinical incidence of lipoma, factors in the differential diagnosis of lipoma and sarcoma. *Acta Ortho Scandinavica* 1983; 54:929–34.

9 Springfield D. Liposarcoma. *Clin Orthop Related Research* 1993; 289:50–57.

10 Kransdorf MJ, Moser RP, Meis JM, *et al.* Fat containing soft tissue masses of the extremities. *Radiographics* 1991; 11:81–106.

11 London J, Kim EE, Wallace S, *et al.* MR imaging of liposarcomas: correlation of MR features and histology *Comp Assist Tomography* 1989; 13:832–5.
